# Codon pairs of the HIV-1 *vif* gene correlate with CD4+ T cell count

**DOI:** 10.1186/1471-2334-13-173

**Published:** 2013-04-11

**Authors:** Maria Clara Bizinoto, Shiori Yabe, Élcio Leal, Hirohisa Kishino, Leonardo de Oliveira Martins, Mariana Leão de Lima, Edsel Renata Morais, Ricardo Sobhie Diaz, Luiz Mário Janini

**Affiliations:** 1 Department of Medicine, Federal University of São Paulo, São Paulo, Brazil; 2 Graduate School of Agriculture and Life Sciences, University of Tokyo, Tokyo, Japan; 3 Institute of Biotechnology, Federal University of Pará, Pará, Brazil; 4 Bioinformatics and Molecular Evolution Laboratory, Department of Biochemistry, Genetics and Immunology, University of Vigo, Vigo, Spain

**Keywords:** HIV-1, Epistasis, APOBEC, Vif, Hypermutation, Positive selection, Co-evolution

## Abstract

**Background:**

The human APOBEC3G (A3G) protein activity is associated with innate immunity against HIV-1 by inducing high rates of guanosines to adenosines (G-to-A) mutations (*viz*., hypermutation) in the viral DNA. If hypermutation is not enough to disrupt the reading frames of viral genes, it may likely increase the HIV-1 diversity. To counteract host innate immunity HIV-1 encodes the Vif protein that binds A3G protein and form complexes to be degraded by cellular proteolysis.

**Methods:**

Here we studied the pattern of substitutions in the *vif* gene and its association with clinical status of HIV-1 infected individuals. To perform the study, unique *vif* gene sequences were generated from 400 antiretroviral-naïve individuals.

**Results:**

The codon pairs: 78–154, 85–154, 101–157, 105–157, and 105–176 of *vif* gene were associated with CD4+ T cell count lower than 500 cells per mm^3^. Some of these codons were located in the ^81^LGQGVSIEW^89^ region and within the BC-Box. We also identified codons under positive selection clustered in the N-terminal region of Vif protein, between ^21^WKSLVK^26^ and ^40^YRHHY^44^ regions (*i.e*., 31, 33, 37, 39), within the BC-Box (*i.e*., 155, 159) and the Cullin5-Box (*i.e*., 168) of *vif* gene. All these regions are involved in the Vif-induced degradation of A3G/F complexes and the N-terminal of Vif protein binds to viral and cellular RNA.

**Conclusions:**

Adaptive evolution of *vif* gene was mostly to optimize viral RNA binding and A3G/F recognition. Additionally, since there is not a fully resolved structure of the Vif protein, codon pairs associated with CD4+ T cell count may elucidate key regions that interact with host cell factors. Here we identified and discriminated codons under positive selection and codons under functional constraint in the *vif* gene of HIV-1.

## Background

The APOBEC (apolipoprotein B mRNA-editing catalytic polypeptide) gene family includes several members, APOBEC1, APOBEC2, APOBEC3 and APOBEC4 that have cytidine deaminase activity [1-3]. Notably, two genes of APOBEC3 (APOBEC3G and APOBEC3F) have been linked to innate immunity, and their ability to restrain retroviral infections has been widely recognized [4,5]. APOBEC3G (A3G) induces cytidine deamination (C→U) in the negative strand of HIV-1 during reverse transcription, hence inducing substitutions of guanosines for adenosines (G→A) in the positive strand of the viral DNA. This mechanism is known as hypermutation and may cause the appearance of stop codons followed by the complete loss of reading frames of the viral genes. HIV-1 counteracts A3G activity through ubiquitination of this host protein through the activity of Vif proteins. Vif proteins assemble with viral-specific E3 ubiquitin ligase through its interaction with cellular Cullin5 (Cul5)-ElonginB-ElonginC proteins, inducing ubiquitination of A3G and consequent degradation by the proteasomal complex [6-10]. Hypermutation is not enough to curb HIV-1 infection because proviruses with varying amounts of G→A mutations are commonly observed in the host genome [11-13]. Furthermore, the polymorphisms in the *vif* gene have been associated with more or less efficacy to neutralize A3G [5]. For these reasons, it has been hypothesized that when G→A mutations are ineffective in neutralizing viral genomes, the side effect is that A3G can actually promote HIV-1 diversification [14,15].

The interaction between the cellular A3G and the *vif* gene of HIV likely emerged from a process of co-adaptation due to recurrent retroviral infections during the evolutionary history of primates [2,16]. Specifically, the repeated encounters with retroviruses probably promoted the fixation of the allelic variants in the genes of the human family of the APOBEC [17-20].

Recently, we found that A3G polymorphisms are mostly unrelated with CD4+ T cell counts of HIV-1 infected Brazilians [21]. Thus, population-based studies may provide conflicting results regarding the overall effect of A3G-vif interactions [5,21-23]. To gain more insights on the A3G-HIV interaction, we used codon-based approaches to determine the function of amino acid substitutions of Vif protein. The study was made through the analysis of 400 *vif* gene sequences obtained from HIV-1 infected drug-naïve individuals.

## Methods

### HIV-1 infected individuals

This study was approved by the Ethics Committee of the Federal University of São Paulo and by the Brazilian Ministry Health; all biological samples were obtained in full accordance with signed informed consent forms.

### DNA samples

Proviral DNA was extracted from heparinized peripheral blood obtained from 400 HIV-1 infected individuals that were drug-naïve (not receiving any antiretroviral therapy) and asymptomatic when samples were collected. From each patient one unique sequence of HIV-1 *vif* gene was generated, then our study focused on the diversity of the virus at the population level. The study group represented almost equally the male (55.5%) and female (45.5%) populations and was composed of three ethnics groups: white (49.2%), mulatto (41.6%) and black (9.2%) individuals. The CD4 counts (cells/mm^3^) ranged from 20 to 5362 and the virus load ranged from 80 to 7.8 x 10^7^ (RNA copies/ml of plasma). HIV-1-infected individuals sampled from São Paulo city between 1989 and 2006 comprised our target population. These individuals were enrolled in the AIDS program of the Brazilian Ministry of Health.

### PCR and sequencing of the vif gene of HIV-1

The vif sequence was amplified by a nested PCR. The primers were designed to cover the entire *vif* gene, according to the reference sequence HXB2 (HIV Sequence Database). The first round was performed with the primers, Platinum Taq DNA Polymerase, 10X Reaction Buffer, MgCl2 (Invitrogen, USA) and deoxyribonucleotide triphosphates (dNTP; GE Healthcare, USA). The second-round PCR was carried out using 5 μl of the first-round product and internal primers. Amplified vif DNA was purified and then sequenced using the BigDye Terminator kit, version 3.1 (Applied Biosystems/Perkin Elmer, Foster City, CA). The samples were electrophoresed on an ABI 3130 genetic analyzer, and the sequencing data were analyzed using ABI software Sequencing Analysis Software.

Sequences Analysis. Nucleotide and protein sequence analyses and edits were performed using the Sequencher DNA Sequence Assembly Software (Gene Codes Corporation, USA).

### Hypermutation detection in the integrase gene of HIV-1

We used a previously described approach to detect the presence of hypermutation in the PCR products of the integrase gene of HIV-1 [24]. Briefly, the PCR products were initially analyzed on 1% agarose gels to confirm amplification. After that, a second electrophoresis was performed with HA yellow (9 μL/mL) incorporated into the agarose gel solution at 65°C and pH 7.5. The electrophoresis was performed at 80 V in 0.5× Tris-borate-EDTA (TBE) for 150 min. The HA yellow gel was visualized after immersion in a solution of ethidium bromide, using the Geldoc-it TS Imaging Systems BioImaging (UVP, Cambridge, CA, EUA). HA yellow is a compound consisting of the DNA ligand, bisbenzamide, covalently linked to polyethylene glycol (PEG) (Resolve-It Kit - Vector Laboratories, Burlingname, CA, USA). Bisbenzamide binds preferentially to AT-rich regions in the DNA and, when coupled to PEG, retards DNA mobility during gel electrophoresis according to the AT content. We used three distinct samples that independently amplified as negative (no hypermutation) and positive controls (hypermutated). The hypermutation statuses of the controls were confirmed by bacterial cloning followed by sequencing.

### Sequence alignment and phylogenetic inference

Initially, the sequences of the vif gene of HIV-1 were aligned using the ClustalX program [25]. Sequences with stop codons and hypermutations were excluded from the analyses. We used the Hypermut software (http://www.hiv.lanl.gov/content/sequence/HYPERMUT/hypermut.html).

After this editing process, the sequences were manually aligned using the SE-AL program, version 2.0 (Department of Zoology, Oxford University; http://evolve.zoo.ox.ac.uk/software/). To construct maximum likelihood (ML) trees, we used the HKY model [26], as implemented in the PhyML software [27]. These trees were used mainly to the selective regimen analysis.

### Association of HIV vif gene and CD4+ cell counts

We investigated whether individual codons or pairs of codons in *vif* gene were associated with levels of CD4+ T cell counts. To do that linear regression and permutation tests were used. The log-transformed CD4 counts were regressed on the amino acids or amino acid pairs. To account for multiplicity, we generated 1000 sets of samples under the null hypothesis of no association by permuting the CD4+T counts. The *p*-values obtained by the log likelihood ratio statistics were contrasted with the null distribution of minimum *p*-values among amino acid positions with SNPs and pairs of these positions.

### Covariation among codons based on phylogenies

A Bayesian Graph method (BGM) was used to explore covariation among amino acids in codons of the *vif* gene taking into account the phylogenetic information of the sequences [28]. Therefore, BGM considers the potential bias due to the founder effect and relaxes the assumption of pairwise associations. BMG reconstructs the maximum likelihood of evolutionary history of the extant sequences, and then it analyzes the joint probability distribution of substitution events among sites in the sequences through a Bayesian graph model. The method was used to detect co-evolving sites in vif. The analyses were performed assuming a GTR model [29], and sites with a marginal posterior probability of 0.85 were considered to be under epistasis. The analysis was performed on the Datamonkey web server (http://www.datamonkey.org).

### Detection of selective pressure

We used a codon-based maximum likelihood method to estimate the selection pressures of the vif sequences. This approach estimates the likelihood of distinct models of codon evolution and computes the ratio (*d*_*N*_*/d*_*S*_=ω) of the number of nonsynonymous (*d*_*N*_) and synonymous (*d*_*S*_) substitution rates between sites considering the phylogenetic relationships of the sequences.

The nonsynonymous/synonymous rate ratio (ω) determines selective pressures at protein level. When selection (neutral) has no effect on the fitness the nonsynonymous and the synonymous mutations will occur at the same rates (*d*_*N*_*=d*_*S*_).

Situations where nonsynonymous mutations are deleterious, negative (purifying) selection will reduce their rate of fixation (*d*_*N*_*<d*_*S*_). If nonsynonymous mutations raise the fitness, their rate will be increased by positive selection (*d*_*N*_*>d*_*S*_).

We used the following codon models. The one-ratio model (M0) assumes a single ω for all sites in the alignment and is the simplest model. The neutral model (M1) allows for different proportions of conserved sites (ω_0_=0) and neutral sites (ω_1_=1), both estimated from the data. Model 1 is the null hypothesis to test for positive selection. The selection model (M2) extends M1 and incorporates an additional class of sites with ω ratios assuming values higher than one (ω_2_ > 1). Significant evidence for positive selection is provided if M2 significantly reject the null hypotheses, M0 and M1, and if the favored models contain a class of codons with ω > 1. Statistical significance can be compared using a standard likelihood ratio test (LRT). These models are implemented in the CODEML program from the PAML v.4 package (http://abacus.gene.ucl.ac.uk/software/paml.html) [30].

## Results

### Diversity of vif gene

To characterize the sequences of the HIV-1 *vif* gene from Brazilians, we analyzed the nucleotide and amino acid substitutions on a site-by-site basis. The overall nucleotide distance of 235 subtype B isolates in the alignment of 581 nucleotides was 0.031±0.004. The translated Vif sequence of 192 amino acids identified 22 singletons, 53 conserved and 138 variable sites. In general the amino acid composition was relatively conserved among subtypes in Brazil and all regions with biological functions were equally conserved. The genetic diversity was estimated assuming the HKY85 model and the analysis were performed using Mega 4.0 software [31].

### Pairs of codons in vif gene associated with CD4+ cells

**Figure 1:**
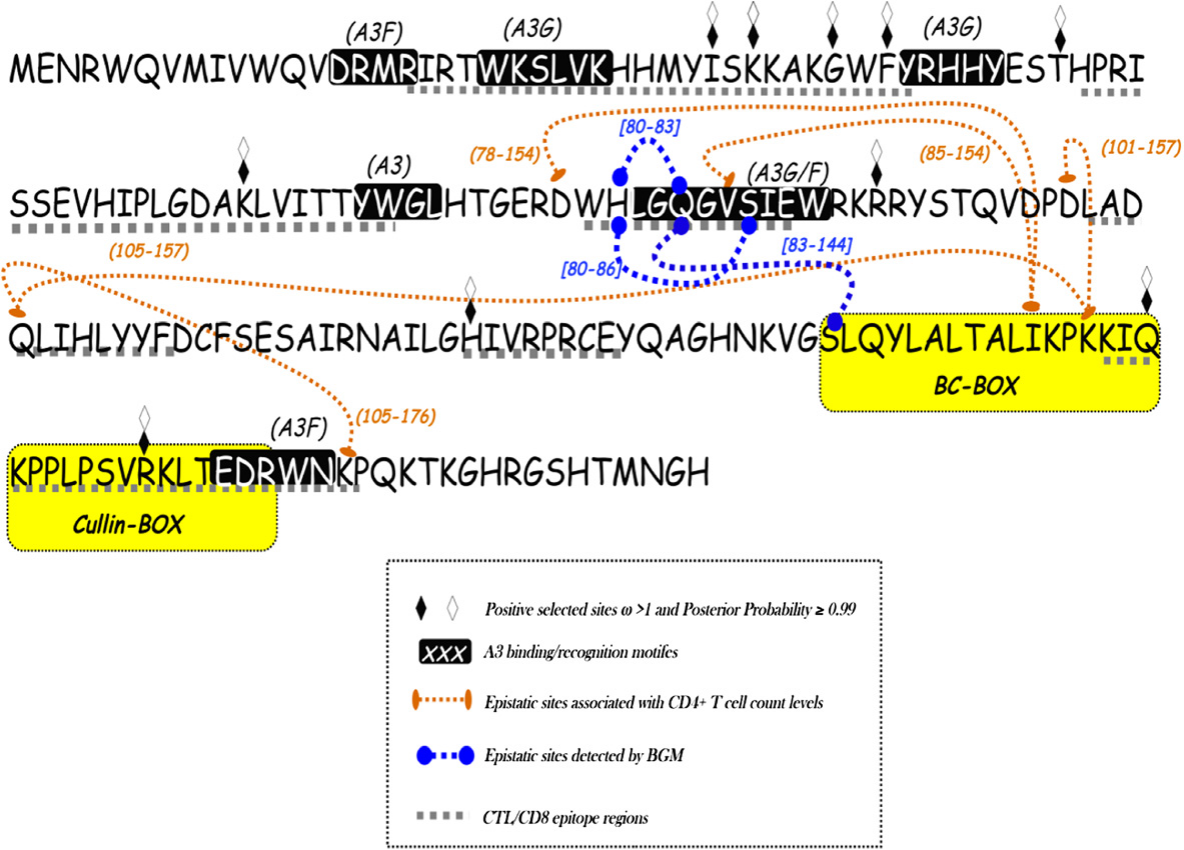
**Consensus sequences of the vif gene of HIV-1.** The consensus was obtained from an alignment of 71 sequences containing only subtype B viruses. Open and filled diamonds indicate sites under positive selection. The figure shows positively selected codons detected in non-recombinant (filled diamonds) sequences and detected in hypermutated viruses (open diamonds). The regions linked to Vif protein functions are highlighted in dark grey with white letters within. The BC-Box and the Cullin5-Box are also highlighted (yellow). Dashed grey lines mark regions where cytotoxic T lymphocytes (CTL) epitopes were previously identified in the Vif protein (http://www.hiv.lanl.gov/content/immunology/maps/maps.html). Orange ovals linked by dashed lines indicate epistatic sites associated with CD4+ cell counts detected by permutation test. Blue circles linked by dashed lines indicate co-evolving sites detected by BGM analysis.

**Figure 2:**
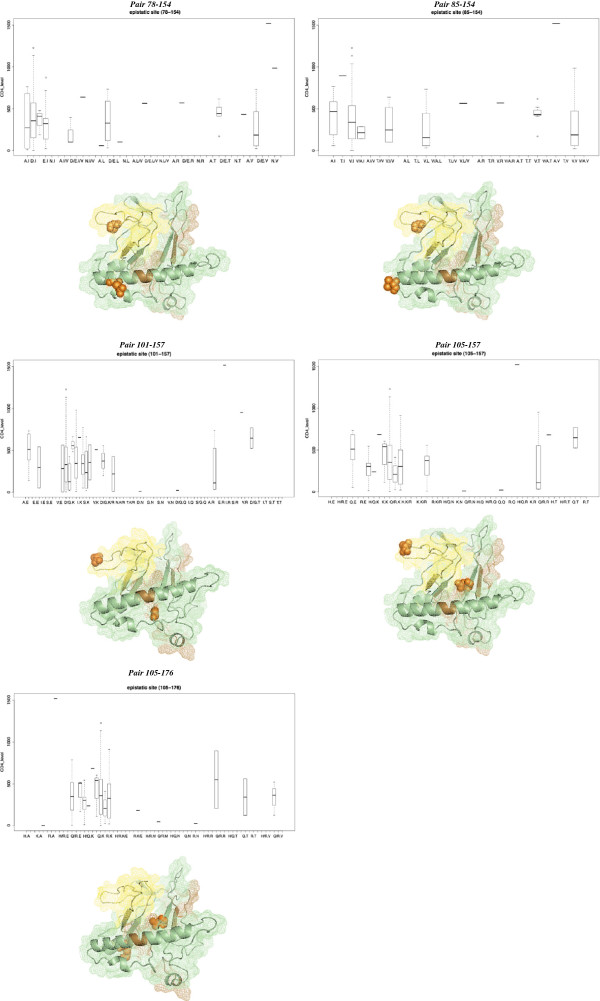
**Epistatic codons and CD4 levels.** Upper panel of each page shows the correlation between epistatic pairs and CD4+ T cell counts. The x-axis depicts the distinct combinations of amino acids (pairs), y-axis shows the levels of CD4+ T cell counts measured as number of cell per mm3 of plasma. Lower panel shows the location of epistatic sites (orange dots) on the structure of a computational model (Balaji et al., Bioinformation. 2006 Dec 6;1:290-309[PMID: 17597910], PDB=1VZF) of Vif Protein of HIV-1. Yellow regions depict the SOCS BOX (BC Box+Cullin Box) and grey regions are A3 binding sites (see text for more details). Visualization and edition of structures were done using PyMOL software (http://www.pymol.org).

**Figure 3:**
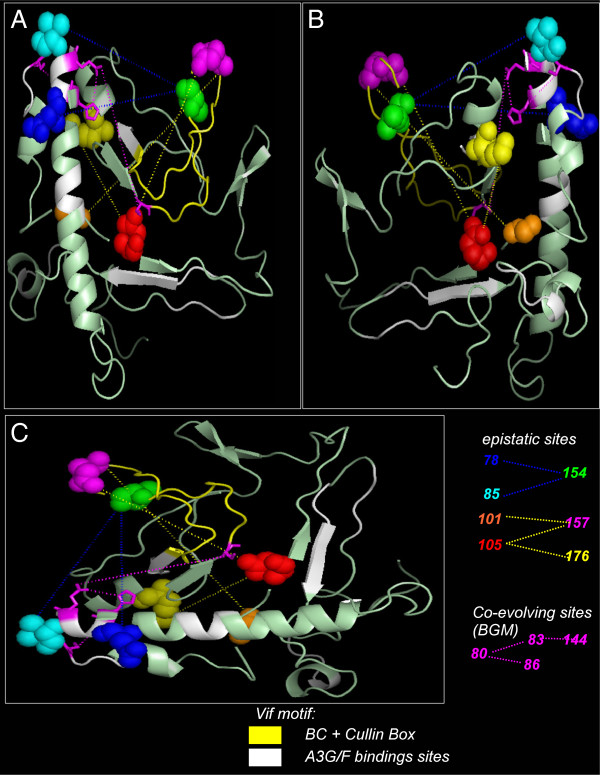
**Epistatic and co-evolving codons in the structure of Vif protein.** 3D structure of a computational model proposed to the Vif protein of HIV-1 (PDB: 1VZF). Epistatic codons are depicted in distinct colors and dashed lines link codon pairs. Co-evolving codons are depicted as magenta sticks in the structure and magenta dashed lines are connecting them. Regions that interact with Apobec3 protein were highlighted in white color in the Vif structure. The BC-Box and the Cullin5-Box were highlighted in yellow color. Panels **A**, **B** and **C** correspond to distinct rotating angles of the structure of Vif.

The regression analysis indicated that no single amino acid positions in *vif* gene were significantly associated with the CD4+T counts. However, when we analyzed the impact of pairs of codons in the levels of CD4+T cells, the epistatic effects of five pairs of amino acids (*i.e*., 78–154, 85–154, 101–157, 105–157, and 105–176 pairs) were detected at a 5% significance level after correction for multiplicity (orange dashed lines in the Figure 1). Notably, most combinations of amino acids in these epistatic sites tend to be associated with CD4+ T cell counts below 500 cells per mm^3^ (see Figure 2 for a detailed description of pairs of residues and their correlation with CD4 counts). We used a proposed three-dimensional computational model of Vif [32] (PDB: 1VZF) to shown the location of the pairs of epistatic codons on the structure of this viral protein (Figure 3).

### Coevolving sites in the vif gene

By using a posterior probability of 0.85, the BGM analysis detected three pairs of codons (*i.e*., 80–83, 80–86 and 83–144) where amino acids were coevolving in *vif* gene. These sites were not the same epistatic sites identified by regression/permutation, although they were concentrated in a specific genomic region of vif between sites 78 to 86 and within the BC-box (see blue dashed lines in the Figure 1 and magenta dotted lines in the Figure 3). However when we reduced the threshold of the posterior distribution to 0.5, various other sites were indicated to be under epistasis, including those identified by the permutation analysis.

### Adaptive mutations in the vif gene

**Figure 4:**
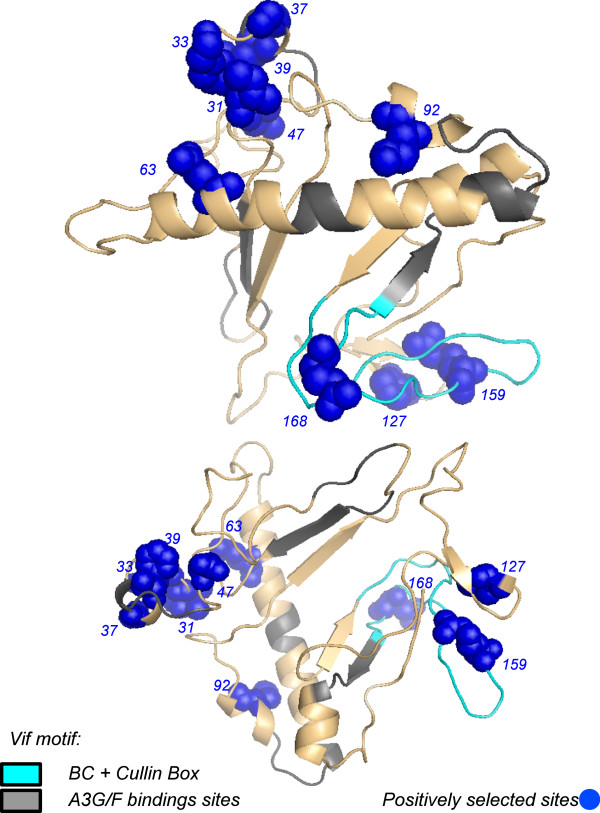
**Positively selected codons in the structure of vif protein.** Codons under positive selection were mapped in structure of Vif protein of HIV-1. Blue spheres indicate locations of positively selected codons. Cyan area designates the BC-Box and the Cullin5-Box. Grey areas indicate regions of Vif that bind to Apobec3 protein.

To explore the selective regimen acting on the *vif* gene of the subtype B lineage of Brazilian isolates, we used a codon-based model to estimate the *dN/dS* ratio. Recombination affects the reliability of likelihood ratio test (LRT) to discriminate models of positive selection [33], then we decided to analyze selective forces in vif sequences that have not recombined. To do that we used a Bayesian approach [34] to identify recombination-free sequences. These sequences were compiled in a data set composed of seventy-one (n=71) recombination-free isolates that were edited in order to exclude sequences with stop codons. The estimated likelihoods of model M2 (−*l*=6882.348) indicated that the hypothesis of positive selection in the evolution of the *vif* gene was effectively accepted to the detriment of the null hypothesis of neutral evolution based on the likelihood of the M1 model (−*l*=6985.462) (χ^2^=206.2277, *p*<0.0001, with two degree of freedom). In detail, the M2 (selection) model detected 57.0% of codons with ω=0.049, 35.5% with ω=1.0 and 7.5% with ω=3.7. Thus, most sites of the *vif* gene evolved under purifying selection. The sites under positive selection (*i.e*., 31, 33, 37, 39, 47, 63, 92, 127, 159 and 168) were mapped onto the consensus of vif sequences from the subtype B lineage (Figure 1). Positively selected sites were distributed along the extension of the gene; codons under positive selection were also detected within regions that have important biological functions, such as the BC-Box and Cullin5-Box (filled diamonds in the Figure 1). In addition, a dataset of vif sequences (n=33) from hypermutated viruses, based on the *integrase* gene, was analyzed to explore the selective regime. According to the M2, most sites (53.6%) evolved under purifying selection with ω=0.035, 37.2% were conserved (ω=1) codons, and 9.2% were under strong positive selection with ω=4.063. These sites under positive selection were exactly the same as those detected in the recombination-free data (open diamonds in the Figure 3). Mapping positively selected sites in the Vif protein sequence and in the 3D structure of a computational model of Vif protein [32] (PDB: 1VZF), revealed they were concentrated between the ^21^WKSLVK^26^ and ^40^YRHHY^44^ motif (*i.e*., 31, 33, 37, 39 and 47), both important to Vif-induced degradation of A3G/F complexes. It is important to mention that the N-terminal region of Vif protein binds selectively to HIV-1 genomic RNA [35] and mRNA of A3G [36]. In addition, within the BC-Box and Cullin5-Box, we also detected codons under high selective pressure (*i.e*., 155, 159 and 168) (Figure 1 and Figure 4). Positively selected sites may indicate adaptive substitutions that usually evolve as a consequence of the host immune response against viral proteins. According to the Los Alamos HIV immunology database, cytotoxic T-lymphocyte (CTL) epitopes have been previously identified in nearly all regions of Vif protein (http://www.hiv.lanl.gov/content/immunology/maps/maps.html/). We then concatenated these CTL epitopes to show them in the consensus sequence of *vif* gene (dotted line in the Figure 1). Our results indicate that the codons under positive selection were not always located within the CTL epitopes, whereas other CTL regions were lacking positively selected codons. Indeed, signatures of positive selection by CTL or antibody immune pressure are a host-specific mechanism that is rarely identified by population-based analyses [37-39].

## Discussion

While hypermutation induced by A3G activity is a natural barrier against retroviruses it is not enough to restrain HIV-1 infection. Sometimes, A3G activity can actually increase HIV-1 diversification [14,15] because G-to-A hypermutation is not always effective to neutralize all viral genomes within a specific host. Our results suggest that the diversity in the HIV-1 *vif* gene is highly associated with adaptation to the host proteins, mainly to increase interaction with cellular components (*i.e.*, elongins and A3G and A3F) to induce APOBEC3 proteasomal degradation.

Particularly, codons under positive selection were more concentrated in a region between the ^21^WKSLVK^26^ and ^40^YRHHY^44^ motifs (*i.e*., 31, 33, 37 and 39). Interestingly, the N-terminal region of Vif protein binds selectively HIV-1 genomic RNA [1] and sites in this region have DNA/RNA binding properties and also interact with A3G/F [35,36]. Additionally, a study showed that the charge of amino acids located between ^21^WKSLVK^26^ and ^40^YRHHY^44^ motifs that are essential for maintaining the ability of vif to bind A3G [40]. Furthermore, it has been shown that the N-terminal region of Vif protein is highly structured, in contrast to the unstructured and flexible C-terminal [41-43]. Likely, the organized N-terminal structure of Vif functions as a connector that binds to A3G/F proteins and DNA/DNA molecules. On the other hand, positively selected sites detected in the C-terminal region of Vif protein were more dispensed. They were found within the BC-Box and Cullin-Box (*i.e*., 127), which both assemble with cellular components to induce A3G proteasomal degradation [6,43-45]. Positive selection was also detected in the vicinity of the PPLP motif (*i.e*., 159), which controls multimerization of Vif proteins [46]. We also found one codon under positive selection (*i.e*., 168) in a region of Vif protein involved in the interaction with Gag, NCp7 and with the cellular membrane [47]. It is worth to note that in the N-terminal region of Vif protein sites under positive selection are clustered between the ^21^WKSLVK^26^ and ^40^YRHHY^44^ motifs whereas in the C-terminal they tend to be dispersed (see Figure 1). Since Vif protein is highly structured at the N-terminal region, contrasting with the unstructured C-terminal [41,42]. Therefore the N-terminal portion of Vif protein tends to be more protected while the C-terminal is solvent exposed and prone to immune recognition. Consequently adaptive evolution in *vif* gene could be related with the host immune surveillance against viral proteins. However, there are various positively selected sites outside CTL epitope regions. Additionally, wide vif sequence intervals in which many CTL epitopes have been empirically detected show no evidence of positive selection. Furthermore, selection driven by antibody evasion or host cell adaptation is rarely detected by population-based analysis [38,48,49]. Indeed, our results showing a distinct pattern of distribution of positively selected sites between N and C terminals of Vif protein mirrors the structural organization of this viral protein. For these reasons, positive selection detected in vif codons likely emerged as an adaptive response to optimize HIV-1 RNA recognition and neutralization of A3G/F in the population.

The comparison of amino acids of vif sequences revealed a limited variability in regions related with A3G/F activity, such as the regions ^14^DRMR^17^ and ^40^YRHHY^44^, which are important for vif-induced degradation of A3G [40,50]. This conservation of vif motifs may indicate a significant evolutionary constraint that has been operating on this viral gene even among distinct lineages. Indeed, we found that most codons (60%) of *vif* gene are predominantly under purifying selection, and perhaps this pattern is needed to preserve its biological function during the viral life cycle. Likewise, HIV-1 *nef* gene is similarly under strong purifying selection [37,51]. Nevertheless, Nef is a multifunctional protein, and this feature can be observed by its plasticity, represented by extensive polymorphism and amino acid length variations that can be detected both in population samples and in the viral population within a single individual as well.

In addition, an attempt was made to establish the influence of the patients’ statuses on the selective regimen of HIV-1. In a previous population-level studies, we observed that CD4+ T cell counts higher than 200 cells/μl were associated with increased *dN/dS* values in the *env* gene of HIV-1 subtype B [48,49,52]. For this reason, we measured the intensity of positive selection in the *vif* gene from datasets categorized into three distinct levels of CD4 counts (>200; 200–400 and <400). We found no difference in the mean *dN/dS* among these data sets (3.65, 3.85 and 3.09 respectively).

Perhaps our most remarkable finding was the identification of five pairs of codons (*i.e*., 78–154, 85–154, 101–157, 105–157, and 105–176 pairs) in the *vif* gene and their association with CD4+ cell levels lower than 500 cells per mm^3^. In each pair (epistatic codons) distinct amino acids combination were associated with distinct levels of CD4+ cells (see Figure 2 for a details). Notably, these pairs of codons were located mainly in the C-terminal of Vif protein (see Figure 1). It has been shown that the mutation ^105^QLI^107^ to ^105^AAV^107^ reduces the infectivity of HIV by 2% [53]. The amino acids between the 154th and 157th positions of the *vif* gene comprise the BC-box, the region that binds cellular elongin B and C to form complexes that trigger the ubiquitination and proteasomal degradation of the A3G proteins [44]. Since codons 154 and 157 are located in the alpha-helix of the BC-box, it is likely that certain amino acids in these sites may affect the interaction with the cellular elongin B and C complex and thereby affect the efficacy of Vif-induced A3G proteasomal degradation. In addition, the ^161^PPLP^164^ motif is fundamental to vif multimerization and interaction with cellular proteins [41,44,46]. Remarkably, proline-to-alanine substitutions in the ^161^PPLP^164^ motif have no effect to the vif structure although it decreased the ability this protein to form oligomers [41]. These findings suggest that domains in the C-terminal of Vif protein fold independently of each other and the flexibility of these domains is required to interact directly with distinct cellular counterparts [41,42]. Thus, we postulate that epistatic effect observed in pairs of codons, indicate electrostatic interaction of certain pairs of amino acids required to Vif activity.

The presence of co-evolving sites was further investigated using a Bayesian graph model that explores associations between codon sites and accounts for the phylogenetic sign of the sequences. The results indicated that amino acids at sites 80–83, 80–86 and 83–144 of vif co-evolve in phylogenies constructed with *vif* gene of the HIV-1. Interestingly, although both methods did not indicate the same sites, these results corroborate the identification of a region between sites 78 to 86 of HIV-1 *vif* gene that has many sites co-evolving with codons located within the BC-box.

## Conclusion

The host-virus interaction between A3G and vif are likely to affect AIDS in many instances. Conversely, the adaptive evolution in the HIV-1 *vif* gene is mainly explained by a response optimized to neutralize A3G activity. Co-evolution detected in some codons suggests that regions of the Vif protein are highly constrained and may have important function to the virus activity. Here, we identified and discriminated codons under positive selection and codons under functional constraint in the *vif* gene of HIV-1.

## Competing interests

The authors declare that they have no competing interests.

## Authors’ contributions

MCB, MLL and ERM performed PCR amplifications and DNA sequencing. SY HK LOM EL executed data analysis. EL HK RSD LMJ participated in the initial design id experiments and evaluated the results. EL HK RSD wrote the manuscript. All authors read and approved the final manuscript.

## Pre-publication history

The pre-publication history for this paper can be accessed here:

http://www.biomedcentral.com/1471-2334/13/173/prepub

## References

[B1] Henriet S Richer D Bernacchi S Decroly E Vigne R Ehresmann B Ehresmann C Paillart JC Marquet R Cooperative and specific binding of Vif to the 5' region of HIV-1 genomic RNA J Mol Biol 2005 354 1 55 72 10.1016/j.jmb.2005.09.025 16236319

[B2] Chiu YL Greene WC The APOBEC3 cytidine deaminases: an innate defensive network opposing exogenous retroviruses and endogenous retroelements Annu Rev Immunol 2008 26 317 353 10.1146/annurev.immunol.26.021607.090350 18304004

[B3] Sheehy AM Gaddis NC Choi JD Malim MH Isolation of a human gene that inhibits HIV-1 infection and is suppressed by the viral Vif protein Nature 2002 418 6898 646 650 10.1038/nature00939 12167863

[B4] Esnault C Heidmann O Delebecque F Dewannieux M Ribet D Hance AJ Heidmann T Schwartz O APOBEC3G cytidine deaminase inhibits retrotransposition of endogenous retroviruses Nature 2005 433 7024 430 433 10.1038/nature03238 15674295

[B5] Pace C Keller J Nolan D James I Gaudieri S Moore C Mallal S Population level analysis of human immunodeficiency virus type 1 hypermutation and its relationship with APOBEC3G and vif genetic variation J Virol 2006 80 18 9259 9269 10.1128/JVI.00888-06 16940537 PMC1563905

[B6] Kobayashi M Takaori-Kondo A Miyauchi Y Iwai K Uchiyama T Ubiquitination of APOBEC3G by an HIV-1 Vif-Cullin5-Elongin B-Elongin C complex is essential for Vif function J Biol Chem 2005 280 19 18573 18578 10.1074/jbc.C500082200 15781449

[B7] Marin M Rose KM Kozak SL Kabat D HIV-1 Vif protein binds the editing enzyme APOBEC3G and induces its degradation Nat Med 2003 9 11 1398 1403 10.1038/nm946 14528301

[B8] Sheehy AM Gaddis NC Malim MH The antiretroviral enzyme APOBEC3G is degraded by the proteasome in response to HIV-1 Vif Nat Med 2003 9 11 1404 1407 10.1038/nm945 14528300

[B9] Yu X Yu Y Liu B Luo K Kong W Mao P Yu XF Induction of APOBEC3G ubiquitination and degradation by an HIV-1 Vif-Cul5-SCF complex Science 2003 302 5647 1056 1060 10.1126/science.1089591 14564014

[B10] Zhang W Chen G Niewiadomska AM Xu R Yu XF Distinct determinants in HIV-1 Vif and human APOBEC3 proteins are required for the suppression of diverse host anti-viral proteins PLoS One 2008 3 12 e3963 10.1371/journal.pone.0003963 19088851 PMC2597746

[B11] Armitage AE Katzourakis A de Oliveira T Welch JJ Belshaw R Bishop KN Kramer B McMichael AJ Rambaut A Iversen AK Conserved footprints of APOBEC3G on Hypermutated human immunodeficiency virus type 1 and human endogenous retrovirus HERV-K(HML2) sequences J Virol 2008 82 17 8743 8761 10.1128/JVI.00584-08 18562517 PMC2519685

[B12] Kijak GH Janini LM Tovanabutra S Sanders-Buell E Arroyo MA Robb ML Michael NL Birx DL McCutchan FE Variable contexts and levels of hypermutation in HIV-1 proviral genomes recovered from primary peripheral blood mononuclear cells Virology 2008 376 1 101 111 10.1016/j.virol.2008.03.017 18436274

[B13] Land AM Ball TB Luo M Pilon R Sandstrom P Embree JE Wachihi C Kimani J Plummer FA Human immunodeficiency virus (HIV) type 1 proviral hypermutation correlates with CD4 count in HIV-infected women from Kenya J Virol 2008 82 16 8172 8182 10.1128/JVI.01115-08 18550667 PMC2519552

[B14] Jern P Russell RA Pathak VK Coffin JM Likely role of APOBEC3G-mediated G-to-A mutations in HIV-1 evolution and drug resistance PLoS Pathog 2009 5 4 e1000367 10.1371/journal.ppat.1000367 19343218 PMC2659435

[B15] Sadler HA Stenglein MD Harris RS Mansky LM APOBEC3G Contributes to HIV-1 Variation Through Sublethal Mutagenesis J Virol 2010 84 14 7396 7404 10.1128/JVI.00056-10 20463080 PMC2898230

[B16] Conticello SG Thomas CJ Petersen-Mahrt SK Neuberger MS Evolution of the AID/APOBEC family of polynucleotide (deoxy)cytidine deaminases Mol Biol Evol 2005 22 2 367 377 15496550 10.1093/molbev/msi026

[B17] Conticello SG The AID/APOBEC family of nucleic acid mutators Genome Biol 2008 9 6 229 10.1186/gb-2008-9-6-229 18598372 PMC2481415

[B18] Di Rienzo A Hudson RR An evolutionary framework for common diseases: the ancestral-susceptibility model Trends Genet 2005 21 11 596 601 10.1016/j.tig.2005.08.007 16153740

[B19] Jern P Stoye JP Coffin JM Role of APOBEC3 in genetic diversity among endogenous murine leukemia viruses PLoS Genet 2007 3 10 2014 2022 17967065 10.1371/journal.pgen.0030183 PMC2041998

[B20] Sawyer SL Emerman M Malik HS Ancient adaptive evolution of the primate antiviral DNA-editing enzyme APOBEC3G PLoS Biol 2004 2 9 E275 10.1371/journal.pbio.0020275 15269786 PMC479043

[B21] Bizinoto MC Leal E Diaz RS Janini LM Loci polymorphisms of the APOBEC3G gene in HIV type 1-infected Brazilians AIDS Res Hum Retroviruses 2011 27 2 137 141 10.1089/aid.2010.0146 20874421

[B22] An P Bleiber G Duggal P Nelson G May M Mangeat B Alobwede I Trono D Vlahov D Donfield S APOBEC3G genetic variants and their influence on the progression to AIDS J Virol 2004 78 20 11070 11076 10.1128/JVI.78.20.11070-11076.2004 15452227 PMC521814

[B23] Do H Vasilescu A Diop G Hirtzig T Heath SC Coulonges C Rappaport J Therwath A Lathrop M Matsuda F Exhaustive genotyping of the CEM15 (APOBEC3G) gene and absence of association with AIDS progression in a French cohort J Infect Dis 2005 191 2 159 163 10.1086/426826 15609224

[B24] Janini M Rogers M Birx DR McCutchan FE Human immunodeficiency virus type 1 DNA sequences genetically damaged by hypermutation are often abundant in patient peripheral blood mononuclear cells and may be generated during near-simultaneous infection and activation of CD4(+) T cells J Virol 2001 75 17 7973 7986 10.1128/JVI.75.17.7973-7986.2001 11483742 PMC115041

[B25] Thompson JD Gibson TJ Plewniak F Jeanmougin F Higgins DG The CLUSTAL_X windows interface: flexible strategies for multiple sequence alignment aided by quality analysis tools Nucleic Acids Res 1997 25 24 4876 4882 10.1093/nar/25.24.4876 9396791 PMC147148

[B26] Hasegawa M Kishino H Yano T Dating of the human-ape splitting by a molecular clock of mitochondrial DNA J Mol Evol 1985 22 2 160 174 10.1007/BF02101694 3934395

[B27] Guindon S Gascuel O A simple, fast, and accurate algorithm to estimate large phylogenies by maximum likelihood Syst Biol 2003 52 5 696 704 10.1080/10635150390235520 14530136

[B28] Poon AF Lewis FI Pond SL Frost SD An evolutionary-network model reveals stratified interactions in the V3 loop of the HIV-1 envelope PLoS Comput Biol 2007 3 11 e231 10.1371/journal.pcbi.0030231 18039027 PMC2082504

[B29] Lio P Goldman N Models of molecular evolution and phylogeny Genome Res 1998 8 12 1233 1244 9872979 10.1101/gr.8.12.1233

[B30] Yang Z PAML 4: phylogenetic analysis by maximum likelihood Mol Biol Evol 2007 24 8 1586 1591 10.1093/molbev/msm088 17483113

[B31] Tamura K Dudley J Nei M Kumar S MEGA4: Molecular Evolutionary Genetics Analysis (MEGA) software version 4.0 Mol Biol Evol 2007 24 8 1596 1599 10.1093/molbev/msm092 17488738

[B32] Balaji S Kalpana R Shapshak P Paradigm development: comparative and predictive 3D modeling of HIV-1 Virion Infectivity Factor (Vif) Bioinformation 2006 1 8 290 309 10.6026/97320630001290 17597910 PMC1891711

[B33] Anisimova M Nielsen R Yang Z Effect of recombination on the accuracy of the likelihood method for detecting positive selection at amino acid sites Genetics 2003 164 3 1229 1236 12871927 10.1093/genetics/164.3.1229 PMC1462615

[B34] Martins Lde O Leal E Kishino H Phylogenetic detection of recombination with a Bayesian prior on the distance between trees PLoS One 2008 3 7 e2651 10.1371/journal.pone.0002651 18612422 PMC2440540

[B35] Bernacchi S Henriet S Dumas P Paillart JC Marquet R RNA and DNA binding properties of HIV-1 Vif protein: a fluorescence study J Biol Chem 2007 282 36 26361 26368 10.1074/jbc.M703122200 17609216

[B36] Mercenne G Bernacchi S Richer D Bec G Henriet S Paillart JC Marquet R HIV-1 Vif binds to APOBEC3G mRNA and inhibits its translation Nucleic Acids Res 2010 38 2 633 646 10.1093/nar/gkp1009 19910370 PMC2810999

[B37] Cavalieri E Florido C Leal E Machado DM Camargo M Diaz RS Janini LM Intrahost and interhost variability of the HIV type 1 nef gene in Brazilian children AIDS Res Hum Retroviruses 2009 25 11 1129 1140 10.1089/aid.2009.0061 19943790

[B38] Lemey P Rambaut A Pybus OG HIV evolutionary dynamics within and among hosts AIDS Rev 2006 8 3 125 140 17078483

[B39] Poon AF Swenson LC Dong WW Deng W Kosakovsky Pond SL Brumme ZL Mullins JI Richman DD Harrigan PR Frost SD Phylogenetic analysis of population-based and deep sequencing data to identify coevolving sites in the nef gene of HIV-1 Mol Biol Evol 2010 27 4 819 832 10.1093/molbev/msp289 19955476 PMC2877536

[B40] Chen G He Z Wang T Xu R Yu XF A patch of positively charged amino acids surrounding the human immunodeficiency virus type 1 Vif SLVx4Yx9Y motif influences its interaction with APOBEC3G J Virol 2009 83 17 8674 8682 10.1128/JVI.00653-09 19535450 PMC2738209

[B41] Bernacchi S Mercenne G Tournaire C Marquet R Paillart JC Importance of the proline-rich multimerization domain on the oligomerization and nucleic acid binding properties of HIV-1 Vif Nucleic Acids Res 2011 39 6 2404 2415 10.1093/nar/gkq979 21076154 PMC3064812

[B42] Marcsisin SR Narute PS Emert-Sedlak LA Kloczewiak M Smithgall TE Engen JR On the solution conformation and dynamics of the HIV-1 viral infectivity factor J Mol Biol 2011 410 5 1008 1022 10.1016/j.jmb.2011.04.053 21763503 PMC3139145

[B43] Stanley BJ Ehrlich ES Short L Yu Y Xiao Z Yu XF Xiong Y Structural insight into the human immunodeficiency virus Vif SOCS box and its role in human E3 ubiquitin ligase assembly J Virol 2008 82 17 8656 8663 10.1128/JVI.00767-08 18562529 PMC2519636

[B44] Donahue JP Vetter ML Mukhtar NA D'Aquila RT The HIV-1 Vif PPLP motif is necessary for human APOBEC3G binding and degradation Virology 2008 377 1 49 53 10.1016/j.virol.2008.04.017 18499212 PMC2474554

[B45] He Z Zhang W Chen G Xu R Yu XF Characterization of conserved motifs in HIV-1 Vif required for APOBEC3G and APOBEC3F interaction J Mol Biol 2008 381 4 1000 1011 10.1016/j.jmb.2008.06.061 18619467

[B46] Yang S Sun Y Zhang H The multimerization of human immunodeficiency virus type I Vif protein: a requirement for Vif function in the viral life cycle J Biol Chem 2001 276 7 4889 4893 10.1074/jbc.M004895200 11071884 PMC1350968

[B47] Wissing S Galloway NL Greene WC HIV-1 Vif versus the APOBEC3 cytidine deaminases: an intracellular duel between pathogen and host restriction factors Mol Aspects Med 2010 31 5 383 397 10.1016/j.mam.2010.06.001 20538015 PMC2967609

[B48] Leal E Janini M Diaz RS Selective pressures of human immunodeficiency virus type 1 (HIV-1) during pediatric infection Infect Genet Evol 2007 7 6 694 707 10.1016/j.meegid.2007.07.008 17719854

[B49] Leal E Casseb J Hendry M Busch MP Diaz RS Relaxation of adaptive evolution during the HIV-1 infection owing to reduction of CD4+ T cell counts PLoS One 2012 7 6 e39776 10.1371/journal.pone.0039776 22768122 PMC3387245

[B50] Russell RA Pathak VK Identification of two distinct human immunodeficiency virus type 1 Vif determinants critical for interactions with human APOBEC3G and APOBEC3F J Virol 2007 81 15 8201 8210 10.1128/JVI.00395-07 17522216 PMC1951317

[B51] Walker PR Ketunuti M Choge IA Meyers T Gray G Holmes EC Morris L Polymorphisms in Nef associated with different clinical outcomes in HIV type 1 subtype C-infected children AIDS Res Hum Retroviruses 2007 23 2 204 215 10.1089/aid.2006.0080 17331028

[B52] Diaz RS Leal E Sanabani S Sucupira MC Tanuri A Sabino EC Janini LM Selective regimes and evolutionary rates of HIV-1 subtype B V3 variants in the Brazilian epidemic Virology 2008 381 2 184 193 10.1016/j.virol.2008.08.014 18809195

[B53] Simon JH Sheehy AM Carpenter EA Fouchier RA Malim MH Mutational analysis of the human immunodeficiency virus type 1 Vif protein J Virol 1999 73 4 2675 2681 10074113 10.1128/jvi.73.4.2675-2681.1999 PMC104023

